# Association Between Consulting Multiple Healthcare Facilities and Medication Burden Among Older Outpatients

**DOI:** 10.7759/cureus.117252

**Published:** 2026-09-30

**Authors:** Hatsuho Ejima, Yuiko Suzuki, Ryo Nonaka, Saki Nakayama, Yurika Hirota, Atsuhiro Kanno, Katsutoshi Furukawa

**Affiliations:** 1 Division of Geriatric and Community Medicine, Tohoku Medical and Pharmaceutical University, Sendai, JPN

**Keywords:** geriatrics, healthcare, medication, pharmacotherapy, polypharmacy

## Abstract

Polypharmacy, which is defined as five or more medications, is a major challenge in older adults and is associated with adverse drug events, poor medication adherence, and increased healthcare utilization. Fragmented healthcare, characterized by consultations with multiple medical facilities, may contribute to medication burden through multiple prescribing and insufficient medication reconciliation. However, quantitative evidence supporting this association remains limited. This study investigated the relationship between the number of healthcare facilities consulted and the total number of medications prescribed among older outpatients. This retrospective observational study included 317 outpatients aged ≥ 65 years who attended Tohoku Medical and Pharmaceutical University Hospital. Data on the number of regularly prescribed medications and the number of healthcare facilities consulted were extracted from electronic medical records. The association between these variables was evaluated using Spearman's rank correlation analysis. Multiple regression analysis was additionally performed to examine whether the association remained significant after adjustment for age. Statistical analyses were conducted using SAS version 9.4 (SAS Institute Inc., Cary, NC, USA), with *P* < 0.05 considered statistically significant. The mean age of the participants was 78.8 ± 15.6 years, and 191 (60.3%) were women. A significant positive correlation was observed between the number of healthcare facilities consulted and the total number of prescribed medications (Spearman's correlation coefficient = 0.39, *P* < 0.0001). Medication burden increased progressively as the number of healthcare facilities increased. The association remained significant after adjustment for age, indicating that age alone did not account for the observed relationship. Consulting multiple healthcare facilities was significantly associated with increased medication burden among older outpatients. These findings suggest that fragmented healthcare utilization may contribute to polypharmacy independently of age. Older adults receiving care from multiple healthcare providers may represent a priority population for pharmacist-led medication reconciliation, comprehensive medication review, and interprofessional care coordination to reduce medication-related problems and optimize pharmacotherapy.

## Introduction

Polypharmacy, which is defined as five or more medications, is an increasingly important public health challenge in aging societies and is associated with adverse drug events, drug-drug interactions, poor medication adherence, hospitalization, and increased healthcare costs [1]. The pooled global prevalence of polypharmacy was reported to be 50.4% [2], and the prevalence of polypharmacy follows the order of Europe, Oceania, North America, Asia, and South America [3]. Although multimorbidity is a major contributor to polypharmacy, fragmentation of healthcare has also emerged as an important factor that may increase medication burden [4]. Older adults frequently consult multiple healthcare providers across hospitals, clinics, and specialized practices, often receiving prescriptions from several physicians. Polypharmacy in older adults is primarily driven by multimorbidity, disease-specific prescribing guidelines, consultations with multiple healthcare providers, prescribing cascades, preventive pharmacotherapy, inadequate medication review, self-medication, and the lack of systematic deprescribing [5,6]. Without effective communication and coordinated medication management, fragmented care may lead to duplicate prescribing, prescribing cascades, and potentially inappropriate medication use [7].

Medication reconciliation and comprehensive medication review are essential strategies for reducing medication-related problems [8,9], and pharmacists play a central role in identifying duplicate therapies, optimizing pharmacotherapy, and improving continuity of care [10]. However, despite the widespread recognition that fragmented healthcare may contribute to polypharmacy, quantitative evidence demonstrating the relationship between the number of healthcare facilities consulted and medication burden remains limited.

We hypothesized that consulting multiple healthcare facilities would be associated with an increased number of prescribed medications among older adults. Therefore, this study investigated the association between the number of healthcare facilities consulted and the total number of prescribed medications in older outpatients. By clarifying this relationship, we aimed to identify a simple indicator that may help healthcare professionals recognize patients at increased risk of medication-related problems and facilitate targeted pharmacist-led medication review and interprofessional care coordination.

## Materials and methods

This retrospective observational study was conducted at Tohoku Medical and Pharmaceutical University Hospital, Japan. Electronic medical records of consecutive outpatients aged 65 years or older who attended the hospital during the study period were reviewed. A total of 317 patients (126 men and 191 women) were included in the analysis. The mean age of the study population was 78.8 ± 15.6 years. Patients with incomplete medication records or insufficient information regarding the number of healthcare facilities consulted were excluded from the analysis.

Demographic and clinical data were extracted from the electronic medical record system from January 1st 2025 to December 31st 2025. The primary variables of interest were the total number of regularly prescribed medications and the number of healthcare facilities consulted by each participant. Healthcare facilities included hospitals, clinics, and other medical facilities providing outpatient medical care. The number of prescribed medications was defined as the total number of regularly prescribed oral, topical, inhaled, injectable and other prescription medications documented at the time of assessment. Temporary medications prescribed for acute illnesses and over-the-counter medications were not included.

The primary outcome was the association between the number of healthcare facilities consulted and the total number of prescribed medications. We hypothesized that consultation with a greater number of healthcare facilities would be associated with a higher medication burden. Because advancing age is closely associated with multimorbidity and polypharmacy, an additional analysis was performed to determine whether age influenced the observed association.

Continuous variables are presented as mean ± standard deviation (SD). The association between the number of healthcare facilities consulted and the total number of prescribed medications was evaluated using Spearman's rank correlation coefficient because the variables were not assumed to follow a normal distribution. Correlation coefficients (ρ) and corresponding P values were calculated.

To examine whether age confounded the relationship between healthcare facility utilization and medication burden, multiple regression analysis was additionally performed with the total number of prescribed medications as the dependent variable and the number of healthcare facilities consulted and age as independent variables. Statistical significance was defined as a two-sided P value of less than 0.05.

All statistical analyses were performed using Statistical Analysis System (SAS) software, version 9.4 (SAS Institute Inc., Cary, NC, USA).

## Results

A total of 317 older outpatients were included in the analysis, comprising 126 men (39.7%) and 191 women (60.3%). The mean age of the participants was 78.8 ± 15.6 years. The number of healthcare facilities consulted and the total number of regularly prescribed medications were extracted from electronic medical records for all participants.

A significant positive correlation was observed between the number of healthcare facilities consulted and the total number of prescribed medications (Spearman's correlation coefficient, ρ = 0.39; P < 0.0001; Figure 1). As the number of healthcare facilities increased, the number of prescribed medications also increased in an approximately linear manner. Patients receiving care from a single healthcare facility generally had a lower medication burden, whereas those consulting multiple facilities tended to receive substantially more medications.

**Figure 1:**
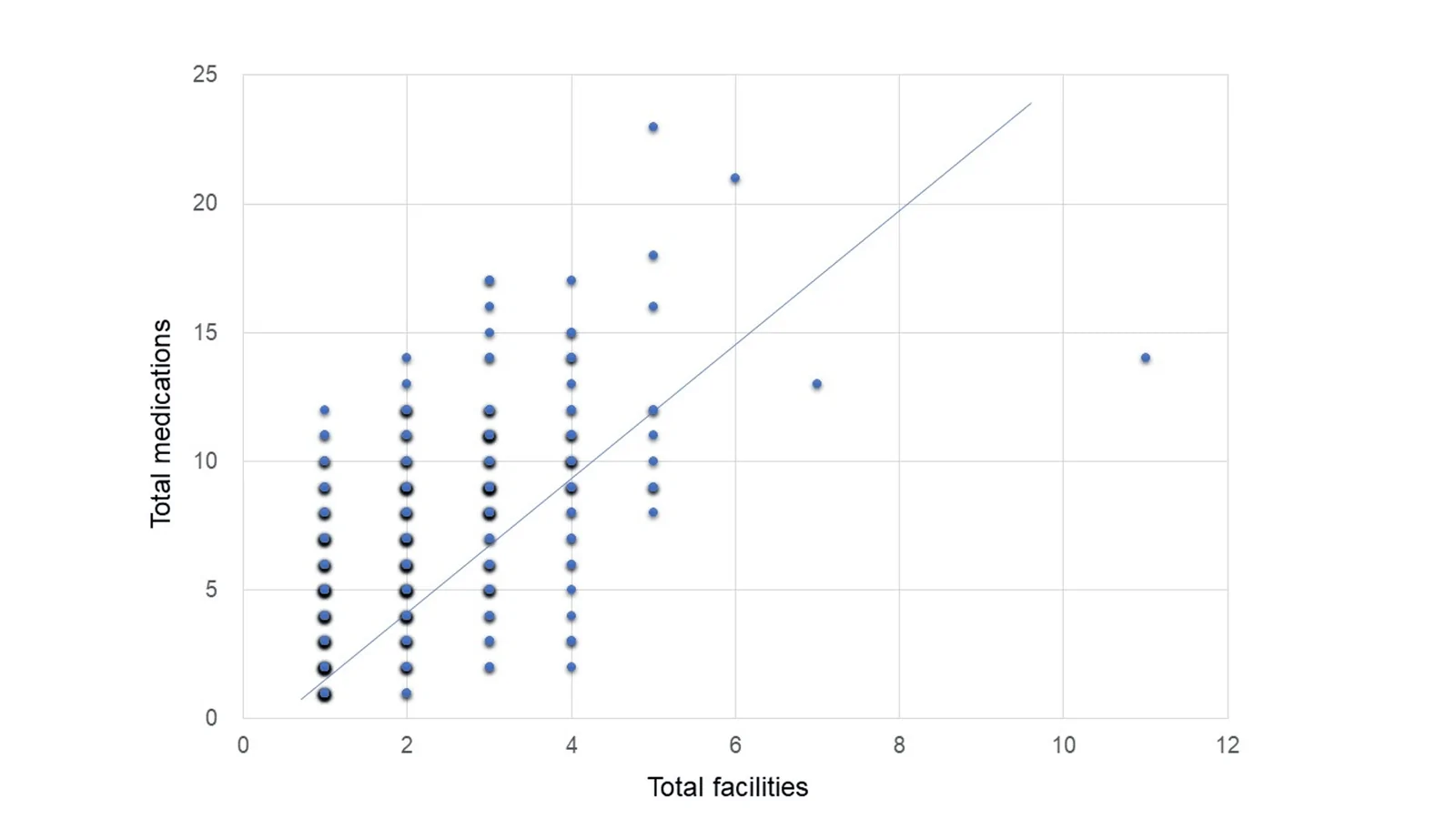
Correlations between the number of medical facilities consulted and the number of medications taken (n=317) The number of medical facilities consulted was plotted against the number of medications taken. A significant positive correlation was observed (ρ = 0.39; P < 0.0001, Spearman rank correlation test).

To determine whether this association was influenced by age, multiple regression analysis was performed with the total number of prescribed medications as the dependent variable and the number of healthcare facilities consulted and age as independent variables (Table 1). As indicated in the table, the association between the number of healthcare facilities consulted and medication burden remained statistically significant after adjustment for age, indicating that the observed relationship was not explained solely by increasing age. On the other hand, age was also a significant determinant of the total number of prescribed medications in the adjusted model. Therefore, both age and medical facilities consulted are independently important factors for polypharmacy in older outpatients. Overall, these findings demonstrate that consulting multiple healthcare facilities was independently associated with a greater medication burden among older outpatients. The consistent positive association suggests that fragmentation of healthcare may contribute to increased medication use irrespective of age, identifying patients who consult multiple healthcare providers as a population that may particularly benefit from medication reconciliation and comprehensive medication review.

**Table 1:** Multiple regression analysis for the total number of prescribed medications as the dependent variable and age and the number of healthcare facilities consulted Both age and the number of healthcare facilities consulted independently affect the number of medications significantly.

	Coefficient (β)	Standard error	95% Confidential Interval	P-value
Age	1.00935	0.0131	0.983674 ~ 1.03502	0.04775
Number of medical facilities consulted	1.0084	0.0018	0.9919 ~ 1.02485	0.0032

## Discussion

This study demonstrated a significant positive association between the number of healthcare facilities consulted and the total number of prescribed medications among older outpatients. Importantly, this relationship remained significant after adjustment for age, suggesting that the increased medication burden was associated with healthcare fragmentation rather than age alone. These findings provide quantitative evidence supporting the widely recognized clinical observation that patients receiving care from multiple healthcare providers are more likely to experience polypharmacy [7,11].

Several mechanisms may explain this association. Older adults frequently have multiple chronic conditions requiring care from physicians with different specialties [8,12]. Although specialist care is essential for optimal disease management, fragmented healthcare may reduce communication among healthcare providers and limit opportunities for comprehensive medication review. Consequently, duplicate prescribing, therapeutic overlap, and prescribing cascades may occur, resulting in an increased medication burden. Previous studies have reported that poor coordination of care contributes to medication-related problems, including adverse drug events and potentially inappropriate prescribing, particularly among older adults with multimorbidity [13,14]. Our findings are consistent with these observations and further suggest that the number of healthcare facilities consulted may serve as a practical indicator of patients at increased risk of excessive medication use.

From the perspective of pharmaceutical care, these findings highlight the importance of medication reconciliation and regular medication review. Pharmacists are uniquely positioned to evaluate the complete medication profile of patients receiving prescriptions from multiple healthcare providers, identify duplicate or unnecessary medications, detect clinically important drug-drug interactions, and collaborate with physicians to optimize pharmacotherapy [15]. Integrated electronic health records and shared prescribing systems may further facilitate communication among healthcare professionals and improve continuity of medication management. Strengthening interdisciplinary collaboration between physicians, pharmacists, and other healthcare professionals may therefore reduce medication-related harm while maintaining appropriate treatment for multiple chronic conditions [16].

This study has several limitations. First, its retrospective, single-center design limits the generalizability of the findings. Second, because the analysis was cross-sectional, causal relationships between fragmented healthcare utilization and medication burden cannot be established. Third, we did not evaluate comorbidities, disease severity, medication appropriateness, or the prevalence of duplicate prescriptions and potentially inappropriate medications, all of which may influence prescribing patterns. Finally, information regarding prescriptions obtained outside the available medical records may have been incomplete.

Despite these limitations, this study provides clinically relevant evidence that consulting multiple healthcare facilities is associated with increased medication burden in older adults. The number of healthcare facilities consulted may represent a simple and readily obtainable marker for identifying patients who could benefit from pharmacist-led medication reconciliation, comprehensive medication review, and coordinated multidisciplinary care. Future multicenter prospective studies are warranted to determine whether interventions targeting patients receiving care from multiple healthcare providers can reduce polypharmacy and improve clinical outcomes.

## Conclusions

Consulting multiple healthcare facilities was significantly associated with increased medication burden among older outpatients. Patients receiving care from multiple providers may benefit from pharmacist-led medication reconciliation, comprehensive medication review, and improved interdisciplinary communication to optimize pharmacotherapy and reduce medication-related problems associated with fragmented healthcare.

## References

[1] Jokanovic N, Tan EC, Dooley MJ, Kirkpatrick CM, Bell JS (2015). Prevalence and factors associated with polypharmacy in long-term care facilities: a systematic review. J Am Med Dir Assoc.

[2] Tian F, Chen Z, Zhang Y, Cai H, Wan X, Zhou F, Zhou Z (2026). Global prevalence, trends, and dose-response associations of polypharmacy in older adults. J Gerontol A Biol Sci Med Sci.

[3] Wang Z, Liu T, Su Q (2024). Prevalence of polypharmacy in elderly population worldwide: a systematic review and meta-analysis. Pharmacoepidemiol Drug Saf.

[4] Nal M, Cömert U, Öztürk M, Bektaş G (2025). Rational use of medicines in the older adult: the role of trust in the health care system. BMC Geriatr.

[5] Beglinger S, Bretagne L, Volery F (2026). Cross-sectional study on factors associated with hyperpolypharmacy and medication adherence in older adults with multimorbidity and polypharmacy. Drugs Aging.

[6] Molist-Brunet N, Sevilla-Sánchez D, Puigoriol-Juvanteny E, Barneto-Soto M, González-Bueno J, Espaulella-Panicot J (2022). Improving individualized prescription in patients with multimorbidity through medication review. BMC Geriatr.

[7] Cho HJ, Chae J, Yoon SH, Kim DS (2023). Factors related to polypharmacy and hyper-polypharmacy for the elderly: a nationwide cohort study using National Health Insurance data in South Korea. Clin Transl Sci.

[8] Sinnige J, Braspenning JC, Schellevis FG, Hek K, Stirbu I, Westert GP, Korevaar JC (2016). Inter-practice variation in polypharmacy prevalence amongst older patients in primary care. Pharmacoepidemiol Drug Saf.

[9] Al-Share QY, Khasawneh RA, Rababa'h AM, Asfar FN, Mohammad YN (2025). Evaluation of medication appropriateness index in cardiovascular outpatient clinic: a cross-sectional study. Curr Pharm Teach Learn.

[10] Okada N (2026). Pharmacist-driven antimicrobial stewardship: evolving roles in clinical practice. J Pharm Health Care Sci.

[11] Kojima T, Mizokami F, Akishita M (2020). Geriatric management of older patients with multimorbidity. Geriatr Gerontol Int.

[12] George T, Manski-Nankervis JA, Klaic M, Kang G, Sudarsanam TD (2024). Interventions in adult patients with multimorbidity in low-income and middle-income countries: protocol for a mixed-methods systematic review. BMJ Open.

[13] Masnoon N, Shakib S, Kalisch-Ellett L, Caughey GE (2017). What is polypharmacy? A systematic review of definitions. BMC Geriatr.

[14] Barker W, Chang W, Everson J, Gabriel M, Patel V, Richwine C, Strawley C (2024). The evolution of health information technology for enhanced patient-centric care in the United States: data-driven descriptive study. J Med Internet Res.

[15] Saboor M, Momtaz YA, Kamrani AA, Sahaf R (2019). Prescription pattern among Iranian community dwelling older adults. Med Glas (Zenica).

[16] Shen J, Yan J, Xie J, Li L (2025). Proposing a novel ABCDEF framework for managing critical illness in geriatrics: challenges and perspectives. Ann Med.

